# Changing Paradigms in the Management of Acute Uncomplicated Diverticulitis

**DOI:** 10.1177/14574969211011032

**Published:** 2021-05-03

**Authors:** A. Chabok, A Thorisson, M. Nikberg, J. K. Schultz, V Sallinen

**Affiliations:** 1 Department of Surgery, Region Västmanland Hospital, Västerås, Sweden; 2 Centre for Clinical Research Uppsala University, Region Västmanland Hospital, Västerås, Sweden; 3 Department of Radiology, Region Västmanland Hospital, Västerås, Sweden; 4 Department of Gastrointestinal Surgery, Akershus University Hospital, Lørenskog, Norway; 5 Department of Abdominal Surgery, University of Helsinki and HUS Helsinki University Hospital, Helsinki, Finland; 6 Department of Transplantation and Liver Surgery, University of Helsinki and Helsinki University Hospital, Helsinki, Finland

**Keywords:** Colonic diverticulitis, acute uncomplicated diverticulitis, antibiotics, surgery, cancer

## Abstract

Left-sided colonic diverticulitis is a common condition with significant morbidity and health care costs in Western countries. Acute uncomplicated diverticulitis which is characterized by the absence of organ dysfunction, abscesses, fistula, or perforations accounts for around 80% of the cases. In the last decades, several traditional paradigms in the management of acute uncomplicated diverticulitis have been replaced by evidence-based routines. This review provides a comprehensive evidence-based and clinical-oriented overview of up-to-date diagnostics with computer tomography, non-antibiotic treatment, outpatient treatment, and surgical strategies as well as follow-up of patients with acute uncomplicated diverticulitis.

## Introduction

Diverticular disease was first described during the 19th century by anatomists who described the development of the disease with inflammation and its complications including the formation of abscesses and fistulae (1–3). The disease was described as a surgical rarity during the 19th century. At the beginning of the 20th century, Bland-Sutton noted that the incidence had risen dramatically between 1910 and 1920 (4). In 1916, Telling and Gruner published a comprehensive description of diverticulosis and diverticulitis (5). Burkitt and Painter drew attention to the rate of diverticulitis during the 1960s and 1970s and reported the dependence of environmental factors and that differences in incidence between countries were associated with their level of economic development (6).

Colonic diverticulosis is a common condition affecting up to 70% of the population in Western countries by the age of 80 (7–9). However only 4%–5% of patients will develop symptomatic disease, most commonly acute diverticulitis, and about 20% of these patients will have complicated diverticulitis (10–14). Thus, acute diverticulitis usually has an uncomplicated course, which is characterized by the absence of organ dysfunction, abscesses, fistula, or perforations. This review provides a comprehensive evidence-based and clinical oriented overview of up to date diagnostics, medical, and surgical treatment as well as follow-up of patients with acute uncomplicated diverticulitis.

## Diagnostics

### Clinical Diagnosis

The most common presentation of patients with diverticulitis is pain in the left lower abdominal quadrant and changes in bowel habits (constipation or diarrhea) with or without fever. These symptoms are non-specific, and the clinical diagnosis of diverticulitis has a sensitivity of only 45%–72% (15–20); however, the diagnostic accuracy of acute diverticulitis may be increased to 86% with a combination of direct left-sided tenderness, absence of vomiting, and a C-reactive protein (CRP) > 50 mg/L (21,22). Common differential diagnoses for acute diverticulitis include appendicitis, colitis, epiploic appendagitis, and cancer.

### Radiology

To confirm the diagnosis and to differentiate uncomplicated from complicated disease, radiological examination is needed. Since the introduction of computed tomography (CT) during the latter part of the 20th century, this modality has taken over as the primary examination method as it has excellent sensitivity for acute diverticulitis, and is rapid and relatively inexpensive to carry out (18,19,23). However, both ultrasonography (US) and magnetic resonance imaging (MRI) are viable alternatives. US is inexpensive and has high spatial resolution and is as sensitive as CT in the hands of an experienced radiologist, with the advantage of delivering no ionic radiation to the patient (24,25). However, the specificity of CT compared to US is higher (96% vs 90%), and US is highly operator-dependent and time-consuming (26). MRI is sensitive to the presence of diverticulitis; however, it is time-consuming, expensive, and susceptible to motion artifacts from the large bowel that can reduce image quality (27). MRI can be used in pregnant patients and has advantages in fistula diagnostics (28).

**Fig. 1.**
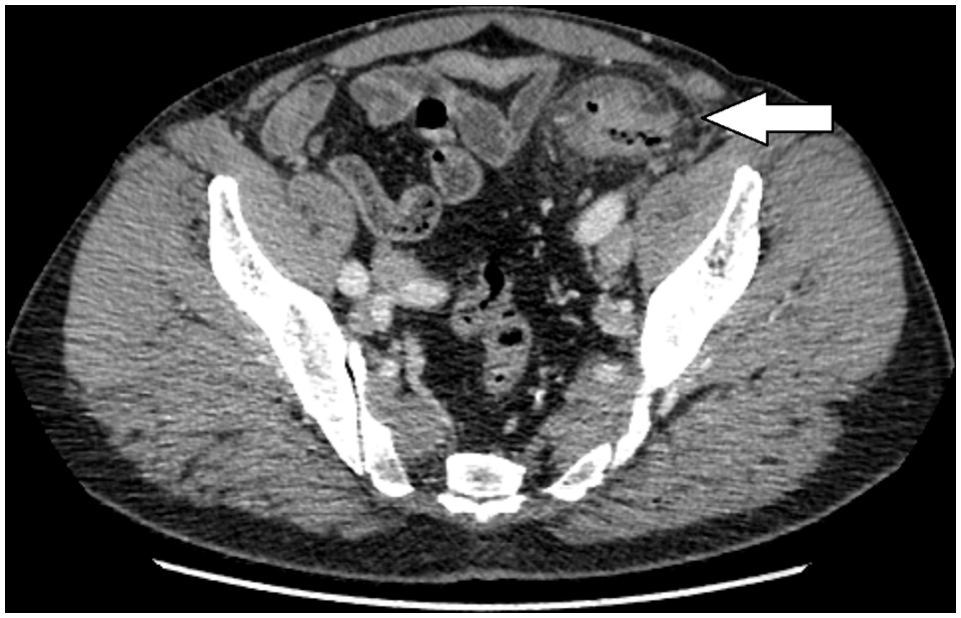
A patient with lower left abdominal pain. This computed tomography scan of the lower abdomen shows colonic wall thickening in the proximal sigmoid colon, diverticula (arrow), and pericolic fat stranding. A diagnosis of uncomplicated diverticulitis was made.

Even though CT is the most common examination tool used for suspected diverticulitis, CT examination protocols (exposure and choice of contrast) differ between countries and hospitals. In most Nordic countries, full-dose CT with intravenous contrast is used (Fig. 1). The use of rectal contrast medium, which is invasive and uncomfortable for the patient, is considered to add limited information in the acute setting. However, it can be advantageous in patients with chronic diverticulitis, especially for visualizing a fistula tract (29). Although low radiation-dose CT without intravenous contrast has a high sensitivity for diverticulitis, smaller perforations and small pericolic or intramural abscesses can be missed using this technique (30). Therefore, a full-dose CT protocol with intravenous contrast is recommended for patients with suspected acute diverticulitis.

### Changes In Treatment Regimens

In the pre-antibiotic era, treatment of diverticulitis consisted of bed rest and no or low residual diet. These treatments had a rather high symptomatic success rate (31). Despite the lack of controlled studies, antibiotics have been used to treat uncomplicated diverticulitis for many years. The reason for this recommendation was the belief that acute diverticulitis is caused by the translocation of intestinal bacteria through the mucosa, resulting in bacterial infection. However, the observation that many patients already showed improvements after one dose of antibiotics and sometimes even before receiving antibiotics raised the question as to whether the improvement was actually in response to antibiotics. This was the background for the first and largest to date randomized controlled trial (RCT; the AVOD study) to evaluate the effect of antibiotics on recovery from acute uncomplicated diverticulitis (32). That study showed that antibiotic treatment neither prevents complications and recurrences nor does it reduce symptoms or length of hospital stay. The findings were confirmed in two other RTCs with patient cohorts from Netherlands and New Zealand/Australia (33,34). In addition, several prospective cohort studies with similar findings, from different countries have been published (Table 1) (20,36,38,39).

**Table 1:** Non-antibiotic therapy in acute uncomplicated diverticulitis.

Study	Year	Number of patients	Design	Outcomes
Chabok et al. (32)	2012	623	RCT	Recovery without complications
Daniels et al.(33)	2017	528	RCT	Time to recovery
van Dijk et al. (35)	2018	468	Follow-up RCT	Complications, recurrence, and surgery
Isacson et al. (36)	2019	556	Follow-up RCT	Complications, recurrence, and surgery
Jaung et al. (34)	2020	180	RCT	Hospital stay
Isacson et al. (37)	2015	155	Prospective	Admission
Mali et al. (38)	2016	161	Prospective	Admission and complications
Estrada et al. (39)	2016	77	Prospective	Complications

RCT: randomized controlled trial; IPDMA: individual participant data meta-analysis.

A long-term follow-up of the AVOD trail with data on 556 patients of the 623 originally included with a mean follow-up time of 11 years showed that antibiotics omittance was safe in the long-term (36). The long-term safety of a non-antibiotic treatment protocol was further confirmed by van Dijk et al. (35) with an analysis of long-term data for patients included in the DIABOLO trial.

Strict patient selection in randomized studies is a drawback, and further studies in a population-based setting are necessary for external validity. Following the findings from RCTs, several retrospective population-based observational studies have shown the implementation and the safety of a non-antibiotics policy for AUD (12,40,41). In the light of this new evidence, several international surgical and gastroenterological organizations and have adopted the non-antibiotic policy (11,42–46). However, gastroenterological organizations in the United State have been more conservative in changing their recommendations (47,48). Interestingly, in a collaboration project between the European and American societies of endoscopic surgery (EAES and SAGES) non-antibiotic policy in AUD was an area of disagreement. Only 26% of the members agreed on the consensus policy and as many as 50% disagreed that the available evidence would change their practice (49). This illustrates that strong evidence alone may not be enough to change traditional treatment habits. Further efforts are needed to convince colleagues around the world that in the absence of septicemia, antibiotics have no place in the management of immunocompetent patients with AUD.

### Inpatient Versus Outpatient Treatments

In recent years, outpatient treatment has gained much attention. In a systematic review, outpatient treatment in selected groups was shown to be safe, reduced healthcare costs considerably, and did not increase the risk of complications, revealing a pooled readmission rate of 7% and very low rates of surgical intervention (50,51). The concept of outpatient treatment without antibiotics was studied for the first time in a prospective cohort study (the PVOD trial) including 155 patients with CT-verified AUD (37). Only four patients (2.6%) were readmitted to hospital because of treatment failure, with none of them requiring surgical intervention. In 2018, Isacson et al. (52) showed that the outpatient regimen for uncomplicated diverticulitis halved the healthcare costs for this patient group with no increased risk of complications. Similarly, in another prospective trial, 140 patients with uncomplicated diverticulitis were treated as outpatients without antibiotics and only four (3%) needed to be admitted to the hospital during follow-up (38). The presented treatment failure rates in the literature vary between 3% and 11% for outpatient treatment (Table 2) (53,54). The first randomized trial on non-antibiotic outpatient treatment of uncomplicated diverticulitis (DINAMO study) presented at the virtual ESCP meeting in 2020 showed similar results to the PVOD trial. However, an outpatient regimen should only be considered in patients with low comorbidity, proven immunocompetence, and the ability to tolerate oral intake.

**Table 2:** Outpatient management for acute uncomplicated diverticulitis.

Study	Year	Number of patients	Design	Antibiotics	Treatment failure rate (%)
Moya et al. (53)	2012	32	Prospective	Yes	6.3
Biondo et al. (54)	2014	132	RCT	Yes	5.3
Isacson et al. (37)	2015	155	Prospective	No	2.6
Mali et al. (38)	2016	140	Prospective	No	2.9
Estrada et al. (39)	2016	77	Prospective	No	11

RCT: randomized controlled trial.

### SURGERY—IS IT NECESSARY?

In 1916, Telling stated in the *British Journal of Surgery* that the treatment of diverticula and diverticulitis “comprised in one word—Surgery” (5). Much has changed since then, and there is now a broad consensus that acute surgery is not indicated in patients with AUD. Even minor complications like small abscesses or covered perforations with extra luminal air can normally be handled conservatively, whereas emergency surgery is mainly reserved to severe complications (bowel obstruction or free perforation with peritonitis) (55,56). Elective sigmoid resection after one or more episodes of uncomplicated diverticulitis has been advocated after two episodes of uncomplicated diverticulitis (57–59). The rational was to prevent complications. However, several studies have shown that the risk of severe complications decreases with the number of diverticulitis episodes ((14,60–63). Consequently, international guidelines have been revised, and there is a consensus that the decision for elective resection should be individualized and not based on the number of previous episodes (49,64). The only legitimate goal of sigmoid resection in an elective setting is to improve the patient’s quality of life. Generally, there are two categories of patients: those with frequent recurrences of AUD and those with ongoing symptoms after an episode of uncomplicated diverticulitis.

There is a variety of mainly retrospective cohort studies investigating elective surgery after uncomplicated diverticulitis, all of which were hampered by a high risk of selection bias (65–69). Fortunately, two RCTs comparing conservative treatment to elective sigmoid resection for recurrent or persistent painful diverticulitis have been published: DIRECT and LASER trials (70,71). The trials’ design was highly similar, but DIRECT trial has published results of 5-year follow-up, while LASER trial has only results for 6-month follow-up. A significant difference in health-related quality of life (HRQoL) favoring surgery was observed after 6 months in both trials, but also at 1 year and 5 years in DIRECT trial (70,72). However, premature abortion of both trials (DIRECT trial due to low recruitment and LASER trial due to benefit in interim analysis) may have led to an overestimation of the effect size (73). Although both trials favor elective sigmoid resection for patients with three or more episodes of diverticulitis within 2-year period, the risks of surgery must be born in mind. Risk for stoma was 5%–21%, and severe complication requiring reoperation occurred in 10%–28% patients randomized to surgery arm (70,71). From an economical point of view and based on DIRECT trial data, elective sigmoid resection was also found to be cost-effective (74). Some limitations of the trials are worth mentioning. Both trials were open-labeled, and a placebo effect in HRQoL results is likely. Although there was minimal (4%) cross-over from conservative treatment to surgery in LASER trial during the first 6 months, significant amount of patients crossed over to surgery in DIRECT trial (23% within 6 months, 46% at 5 years), which means that the results must be interpreted with caution. Key studies on elective sigmoid resection after uncomplicated diverticulitis are summarized in Table 3 (65–72).

**Table 3:** Elective surgery after acute uncomplicated diverticulitis.

Study	Year	Design	Main limitations	Main conclusions
LASER (71)	2020	RCT	Under-powered	Favors surgery
DIRECT trial primary outcome: long-term results (70,72)	2017, 2019	RCT	Under-powered Limited representativeness	Favors surgery
Polese et al. (68)	2018	Retrospective parallel group	Selection bias	Favors surgery
Brandlhuber et al. (66)	2018	Retrospective parallel group	Selection bias	Favors conservative treatment
von Strauss Und Torney et al. (69)	2017	Cross-sectional cohort	HRQoL not evaluated, based on administrative data	Decline in proportion of colon resections over time
Boostrom et al. (65)	2012	Retrospective single cohort	No control group	Improved symptoms after surgery
Klarenbeek et al. (67)	2010	Retrospective parallel group	Selection bias HRQoL not evaluated	Favors conservative treatment for majority of patients

RCT: randomized controlled trial; HRQoL: health-related quality of life.

Any decision on sigmoid resection in patients with AUD should be individualized, and the advantages of elective sigmoid resection, namely superior HRQoL, lower pain, and fewer recurrences, need to be balanced against the significant risk of major complications of surgery.

### Surgical Techniques

Although evidence is limited, currently elective sigmoid resection with primary anastomoses is usually performed with minimal invasive techniques (75). Hartmann’s procedure or temporary diversion of colonic anastomoses is preserved in patients with severe comorbitity or in cases with anastomotic complications. The main advantages of the laparoscopic approach are faster recovery, reduced wound infection rates and a reduced frequency of hernias; however, the conversion rates to open surgery is around 13% (76,77). Robotic sigmoid resection compared to laparoscopic resection might result in lower conversion rates of around 8% (77). More controversial is the extent of colonic resection and whether a central vessel ligation should be performed. Based on retrospective cohort studies, it is widely recommended that the lower resection margin should be at the colorectal junction and that all macroscopically inflamed bowel should be removed (78,79). However, there is no evidence for the removal of all diverticula-bearing proximal colon. Furthermore, there is no rationale for central vessel ligation in diverticular disease when malignancy has been ruled out. As central vessel ligation bears a theoretical risk of impaired perfusion of the colorectal anastomosis and a risk of nerve damage, it is not generally recommended. However, the evidence for this recommendation is sparse (80).

### Follow-Up

There is no consensus in the literature with regards to the need for a routine colonic examination after an episode of AUD. Meta-analyses of studies on cancer prevalence after an episode of acute diverticulitis have shown varying results with a prevalence of malignancy of 0.5%–2% for uncomplicated diverticulitis and 7.9%–10.8% for complicated diverticulitis (81,82). Given the high rate of carcinoma in patients diagnosed with complicated diverticulitis, all patients treated non-surgically for complicated diverticulitis should undergo a colonic examination to rule out malignancy. In patients with CT-verified uncomplicated diverticulitis, the prevalence for colorectal cancer is similar to predicted prevalence in screening populations of similar age in the majority of studies (32,83–88). However, some studies have found higher cancer prevalence in patients with diverticulitis, making omission of routine follow-up colonoscopy difficult (20,89).

In our opinion, omission of a routine follow-up colonoscopy could be considered in patients with CT-verified uncomplicated diverticulitis, where the CT scans have been re-evaluated by a gastrointestinal radiologist, the patient has no sign of colorectal cancer such as anemia, haematochezia, or change in bowel habit and where the symptoms of diverticulitis have diminished at a 4-week follow-up.
